# Long-term evaluation of treatment protocols for isolated midfacial fractures in a German nation-wide craniomaxillofacial trauma center 2007–2017

**DOI:** 10.1038/s41598-021-97858-4

**Published:** 2021-09-14

**Authors:** Lars Bonitz, Vivienne Wruck, Elena Peretti, Dietmar Abel, Stefan Hassfeld, Ákos Bicsák

**Affiliations:** 1 Clinic for Cranial- and Maxillofacial Surgery, Regional Plastic Surgery, Dortmund General Hospital, Chair of the University of Witten-Herdecke, Muensterstrasse 240, 44145 Dortmund, Germany; 2 Health Faculty, University of Witten/Herdecke, Alfred-Herrhausen-Strasse 45, 58453 Witten, Germany

**Keywords:** Dentistry, Diagnosis, Public health, Oral anatomy, Epidemiology, Outcomes research

## Abstract

An update on the trends in maxillofacial traumatology provides additional information on the actual and changing needs. This retrospective study aimed to review all patient records of patients treated for isolated midface fractures in the Department of Cranial- and Maxillofacial Surgery at the Dortmund General Hospital between 2007 and 2017. The patient radiographs and patient files were reviewed. The safety and efficacy of the applied methods were controlled by assessing complications based on the Clavien-Dindo classification system. The statistical analysis included descriptive methods including regression analysis and χ^2^-test. In eleven years, 3474 isolated midface fracture sites have been identified in 2868 patients. The yearly trend is slightly increasing, in elderly clearly worsening, in children and youth decreasing. The male-to-female ratio was 2.16:1 for the whole study population, in the age group 18–25 y.o. 6.95:1 while in elderly above 80 y.o. 1:2.51, the age group specific incidence reflects this result, too. The most common fractures were nasal bone fractures (1405), zygomatic fractures (832) and orbital floor fractures (700). The average hospital stay was 2.7 days, the most fractures were operated within 24 h. The complication rate was 2.02% (Clavien-Dindo class II–V). The incidence of midfacial fractures is increasing in the total population and especially in elderly, but decreasing in children. Development of injury prevention measures is needed in this population. The diagnostic and therapeutic procedures are appropriate, as there is a low complication rate and short inpatient stay observed.

## Introduction

The head and face are exposed to injuries. Facial fractures are common injuries as results of road traffic accidents, falls, interpersonal violence, sports or work-related accidents^1–7^. The most common fractures are nasal bone fractures^6^. Upper jaw fractures, on the other hand, are common in high-energy trauma, therefore often as part of panfacial fractures^8^. The number of cases of isolated fractures of the central midface (except for the isolated nasal bone fractures, which occur most frequently) is reported in these papers as significantly lower.

The development of diagnostics and treatment of midface fractures have been an important topic since 1895 the introduction of the first osteosynthesis system by Lane^9^ and 1896 the first dental radiograph by Walkhoff^10^. After decades of development, computed tomography (CT) became the “gold standard” for diagnostics, even three dimensional virtual applications are used after 2000^11,12^. Different osteosynthesis materials, methods and implant designs have been introduced^13^. Titanium alloy implants have proven their superiority in osteosynthesis^14–16^. The latest improvements include digital workflows, usage of finite element methods and individual implant planning that allows more accurate diagnostics and osteosynthesis in maxillofacial surgery^12,17,18^.

Patient safety, treatment outcome and economic factors have become of increasing importance during these decades^16,19–22^. The complication rates after facial bone fractures are reported between 4.67 and 17%^22^. These have a negative impact on the length of hospital stay, operations in cases with complications generally take longer and patients are longer disabled and need much rehabilitation efforts to be able to return to normal life activites^22^. The removal of the osteosynthesis implants is a matter of discussion^23^, however, if a removal is indicated or requested by the patient, the best time to avoid implant osteointegration is 4–6 months time after surgery^22^.

Based on the continuously evolving knowledge, different fracture treatment guidelines have been established. The current and widely accepted fracture classification and treatment system is presented by the AO (AO Foundation, Clavadelerstrasse 8, 7270 Davos, Switzerland)^24–26^. This is the most comprehensive consensus guidline that has been implemented in maxillofacial traumatology. If these guidelines are correctly applied, the quality of patient care is good. This workflow is supported by good economic decisions and adequate management of patients and resources^19,20,27–30^. The continuous review of the treatment outcome provides feedback about treatment quality.

**Figure 1:**
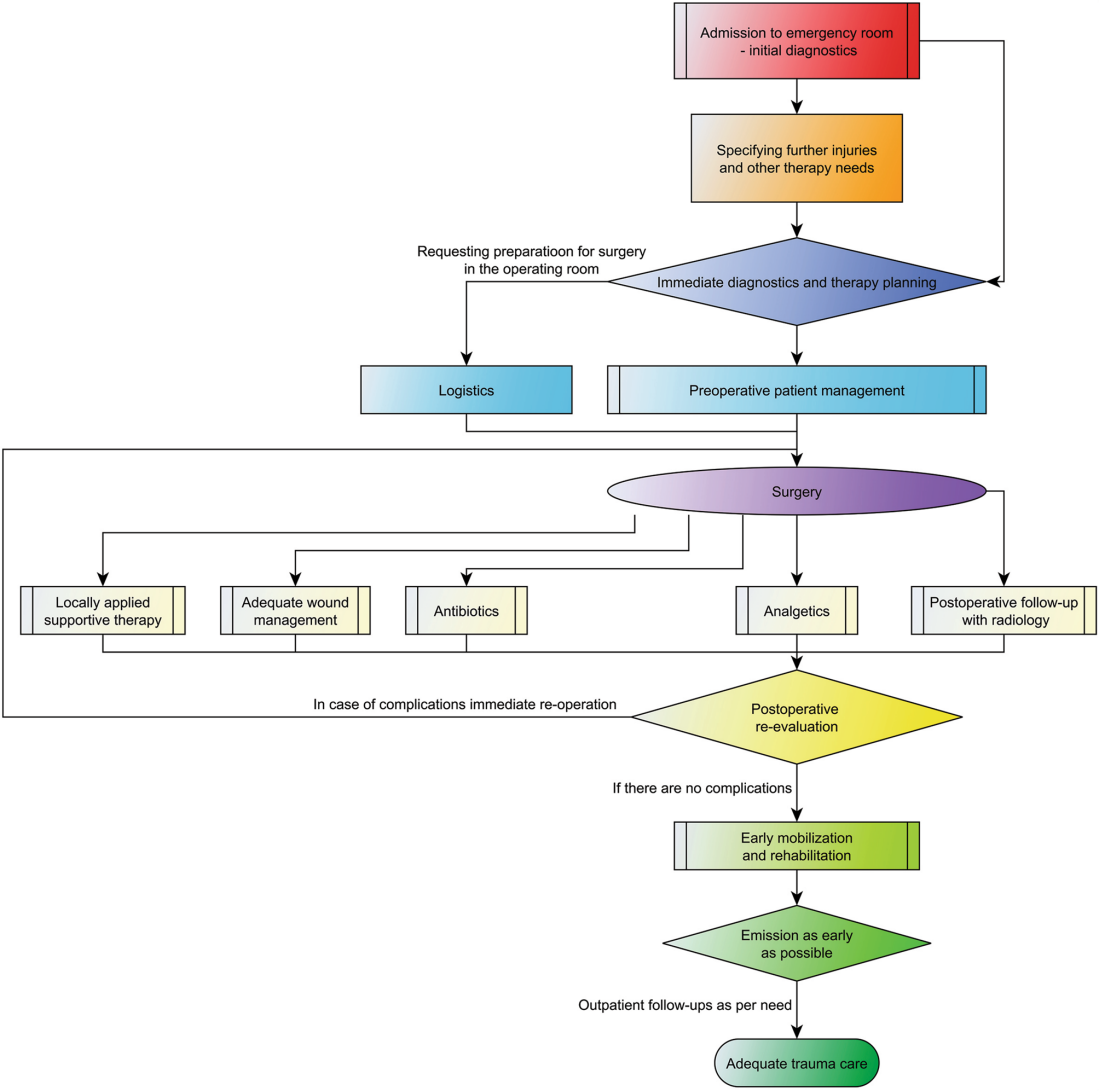
Treatment protocol and workflow used at the Department of Oral- and Maxillofacial Surgery at Dortmund General Hospital.

Figure 1 represents the summary of the workflow applied at the Department of Oral and Maxillofacial Surgery at Dortmund General Hospital. This workflow is based on actual literature and guidelines and is updated regularly. Patients are initially seen in the Outpatient Department of the Clinics or in the Interdisciplinary Emergency Room. After the initial assessment of injuries, a complex diagnostic procedure is completed to determine the therapeutic needs. After this, a treatment plan is prepared and the surgical therapy is carried out as soon as possible. Postoperatively antibiotics is administered (ampicillin-sulbactam 2 g/1 g iv. 3 times a day or clindamycin 600 mg 3 times a day in case of allergies), analgesics is adapted as per WHO-guidelines, cryotherapy is administered. The wound management is performed by trained personnel. In case of need, an early logopedic treatment or physiotherapy are applied. If needed, a further treatment in a rehabilitation unit is initiated. The emissions take place at least after 2 days after surgery, but this period is prolonged individually, if needed. Clinical examinations and radiographs are performed postoperatively. If a complication is diagnosed, the necessary therapy is addressed.

Postoperative follow-up visits are planned mostly 7–10 days after surgery. After less severe injuries, most patients are referred to a follow-up visit to the general practitioner’s or maxillofacial surgeon at the office. Complicated cases or patients with complications are referred to our Outpatient Department. Late complications are addressed in this phase of treatment as per need. As many follow-up visits take place, as needed. The removal of osteosynthesis implants (except for orbita meshes) is planned after 4–6 months time.

Our study aimed to retrospectively assess the safety and efficacy of the current treatment protocols and osteosynthesis procedure by analyzing patient cases with isolated midfacial fractures, who received inpatient care for their injuries at the Department for Oral- and Maxillofacial Surgery and Facial Plastic Surgery at General Hospital Dortmund between 01.01.2007 and 31.12.2017.

## Materials and methods

The Ethics Commission of the University of Witten–Herdecke has approved this study (No. 152/2017).

This study was conducted in accordance with the Helsinki Declaration, the laws and regulations of the European Union, the Federal Republic of Germany, the State North-Rhine-Westfalia and the General Hospital Dortmund.

This study included patients with isolated midface fractures, who have been treated in our department from 1.1.2007 to 12.31.2017. All patients that had any other associated facial fractures have been excluded from this study. The initial patient examination is performed by the attending maxillofacial surgeon. If this is required, a close cooperation with the trauma surgeon, neurosurgeon or other specialities are round the clock available. If the attending surgeon is not yet a specialist, a specialist support is also always available. Patients after admission are reviewed, the diagnosis and treatment plan are discussed and corrected, if needed.

Second, third and fourth Author have reviewed patient documentation, last Author has provided support and has monitored the data collection with an overall source data verification rate of 44% and 100% for cases with complications.

Patients with midface fractures are seen in the outpatient department or in the emergency room (out of office hours). Patients are primarily admitted to the hospital. The diagnostics is performed immediately and the decision for the treatment option is taken as soon as the diagnostic results are available. Patients are diagnosed and operated—in case of dislocated fractures—based on the AO criteria^24,25^. Antibiotics (amoxicillin-clavulanic acid or clindamycin) are administered peri- and 3 days postoperatively, pain medication and cryotherapy is proven. In case of orbital involvement, an examination by eye specialists is mandatory. Patients are regularly discharged 2 days after surgery. The removal of the osteosynthesis implants is mostly offered for the patients to reduce late complications that could result from a foreign body. In these cases, osteosynthesis implants are standard titanium alloy materials that have been marketed in 2003.

The below list contains the fracture sites that have been assessed in this study.

- Fractures of the central midface
  - Nasal bone fracture
  - Naso-orbito-ethmoid fracture (Markovitz Type I, II and III)
  - Medial orbital wall (orbito-ethmoid) fracture
  - Le Fort I type fracture
  - Le Fort II type fracture
  - Palatal fracture
  - Isolated fracture of the anterior sinus wall
- Fractures of the lateral midface
  - Orbital floor fracture
  - Zygomatic arch fracture (only isolated cases)
  - Zygomatic bone fracture
  - Le Fort III type fracture
  - Orbital roof fracture

The complications are evaluated as per the classification of Clavien-Dindo^31^. Class I complications have not been evaluated. Any Class II–V complications were identified in patient documentation and were analyzed. Safety was assessed based on the WHO Guidelines for Safe Surgery. If an adverse consequence (complication, permanent disability) was detected, the procedure is considered as unsafe. In contrary, a completed treatment without complications or permanent disability is considered as safe^32^.

The main study hypothesis was that adopting the latest improvements of maxillofacial traumatology and supporting specialties leads to improvement in complication rate and other measures of care. Further hypothesis was that shortening of the time frame between injury and surgery decreases the rate of complications.

Based on the electronic data, the Dortmund Cranio-Maxillofacial Trauma Registry was created. The suitable patients have been identified in this databank. The radiographs, discharge and surgery reports of these patients have been analyzed. The data were analyzed with Microsoft Excel 2013 (® Microsoft Corp., Redmond, USA). The statistical analysis included descriptive methods including regression analysis and χ^2^-test to verify significance in case of gender and age differences. The confidence interval was *p* < 0.05.

### Ethical approval

The Ethics Committee of the University of Witten–Herdecke has approved this study (No. 152/2017).

### Informed consent for study participation and publication

Informed consent was obtained from all individual participants included in the study.

## Results

**Figure 2:**
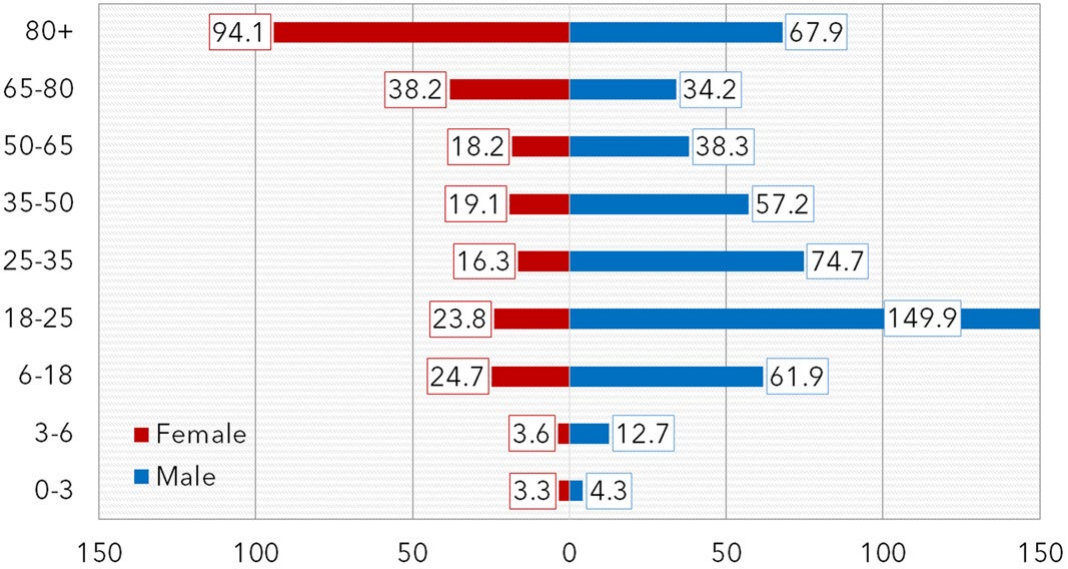
Yearly variance of the number of patients admitted to hospital with midface fracture.

**Figure 3:**
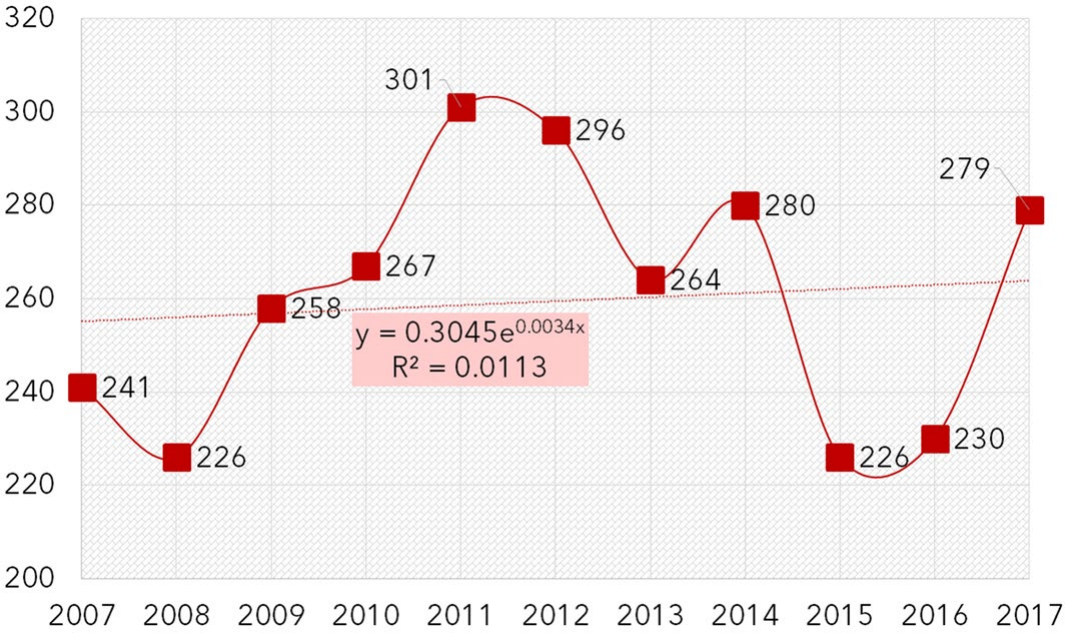
Yearly average incidence of midface fractures in different age groups. (The incidence is given N/100,0000/year, the reference is the city population given in Table 1).

In eleven years from 1.1.2007 to 12.31.2017, a total number of 2868 patients have been admitted to our Department with isolated midface fractures (this makes 40.9% of the 7010 total hospitalizations for maxillofacial traumas). Table 1 reports the demographic data of the patients. A total of 1961 males and 907 females have been admitted (respectively 68.4% and 31.6% of isolated midface cases), the overall male-to-female ratio was 2.16:1, however, this rate shows a high variance between 6.95:1 in 18 to 25-year-old males and 1:2.54 in female older than 80 years old. The yearly number of patients is shown in Fig. 2, the trend is stable. To avoid bias resulting from differently sized population groups, the yearly average incidence rates have been counted based on the city population report^33^. The incidence is the highest in 18–25-year-old males and is generally higher in males before the age of 65 years. In the elderly (65 y.o. and above), the incidence is higher in females (Fig. 3).

**Table 1:** Demographic data.

Age	Number of patients				Male-to-female ratio	*p* value	Yearly average incidence			City population (average)		
	Male	Female	Total	% Total (%)			Male	Female	Total	Male	Female	Total
0–3	4	3	7	0.2	1.33:1	0.593	4.3	3.3	3.8	8482	8253	16,735
3–6	11	3	14	0.5	3.66:1	*0.002*	12.7	3.6	8.2	7894	7636	15,530
6–18	226	84	310	10.8	2.69:1	< *0.001*	61.9	24.7	44.0	33,211	30,911	64,122
18–25	459	66	525	18.3	6.95:1	< *0.001*	149.9	23.8	90.0	27,846	25,169	53,015
25–35	373	74	447	15.6	5.04:1	< *0.001*	74.7	16.3	46.9	45,418	41,314	86,732
35–50	387	122	509	17.7	3.17:1	< *0.001*	57.2	19.1	38.7	61,486	58,191	119,677
50–65	263	126	389	13.6	2.08:1	< *0.001*	38.3	18.2	28.2	62,484	62,784	125,268
65–80	145	195	340	11.9	1:1.34	< *0.001*	34.2	38.2	36.4	38,539	46,467	85,006
80+	93	234	327	11.4	1:2.51	< *0.001*	67.9	94.1	84.8	12,456	22,609	35,065
Total	1961	907	2868	100	2.16:1	< *0.001*	146.3	66.5	106.0	297,816	303,334	601,150

The *p* value refers to the gender difference (*χ*^2^-test, confidence interval 95%, significant values in Italics). In the study time frame, the city population was relatively stable with a yearly change of ± 1.000, thus the report from 2017 was taken as the basis.

The analysis of the etiologies revealed that falls, interpersonal violence, road traffic accidents, sport and free time-related accidents and work-related accidents are the most common causes. Alcohol usage is very common, lately the use of drugs is an emerging issue.

The analysis of fracture sites (Table 2) has shown that the most exposed areas are the nasal bone, the zygomatic bone and the orbital floor (respectively 1405, 832 and 700 fracture sites). Also, in the case of zygomatic bone fractures and orbital floor fractures, there is a significant difference in the number of left and right-sided fractures. This difference results of the high number of interpersonal violence as a cause for these fractures, especially among young males. Classic Le Fort-fractures (a total of 178 in all three levels) or fractures in the nasoethmoid regions (a total of 53 fracture sites) are in comparison less common. Differently from the referred AO-classification, where this is not listed—in 40 cases, we found isolated fracture of the frontal maxillary sinus wall resulting mostly from falls or interpersonal violence.

**Table 2:** List of the fracture sites.

	Left	Right	Total	% of midface fractures (%)	*p* value
Nasal bone	x	x	1405	40.4	
Zygomatic bone	471	361	832	23.9	< 0.001
Orbital floor	394	306	700	20.1	< 0.001
Zygomatic arch, isolated	85	67	152	4.4	0.038
Le fort I	60	56	116	3.3	0.599
Le fort II	34	31	65	1.9	0.599
Dentoalveolar	x	x	61	1.8	
Maxillary sinus wall	14	26	40	1.2	0.007
Orbital roof	14	21	35	1.0	0.094
Naso-orbito-ethmoid, Type II	9	11	20	0.6	0.527
Naso-orbito-ethmoid, Type I	8	6	14	0.4	0.449
Orbito-ethmoid	6	6	12	0.3	1
Le fort III	3	4	7	0.2	0.593
Naso-orbito-ethmoid, Type III	4	3	7	0.2	0.593
Other upper jaw	4	1	5	0.1	0.058
Hard palate	2	1	3	0.1	0.414
Total			3474		

The *p* value refers to the difference between the left and right-sided fractures (χ^2^-test, confidence interval 95%). In the column [%] of midface fractures the proportion of fractures in a region in relation to all midface fractures are shown in percents.

**Figure 4:**
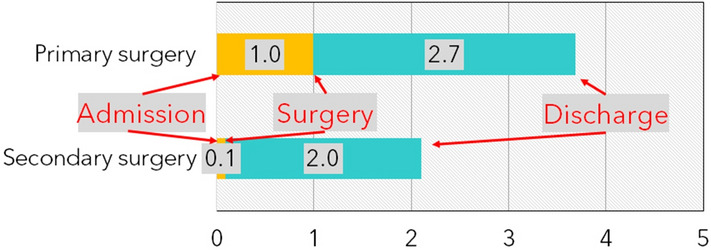
The average duration from admission to surgery and from surgery to discharge (in days), for primary and secondary surgeries (values rounded).

The surgical treatment is performed mostly within 24 h after the injury (Fig. 4), 1.0 (SD = ± 0.3) days in case of first surgeries and 0.1 (SD = ± 0.7) days in case of secondary surgeries. The patients are observed 2.7 (SD = ± 4.9) days in case of first surgeries and 2.0 (SD = ± 0.7) days after secondary surgeries thereafter until discharge. The average surgery in the central midface area took 21 (SD = ± 26.2) minutes, in the lateral midface area 33 (SD = ± 27.4) minutes. Respectively the average anesthesia durations were 63 (SD = ± 78.6) and 90 (SD = ± 67.7) minutes. This grouping was performed to differentiate between different surgical technics applied in the nasal and periorbital regions.

Table 3 provides a summary of the most frequently used osteosynthesis implants. The 2.0 mm straight plate without bar is mostly used (37.6%), this one is used in many zygomatic bone fractures (23.9% of all fractures) at the frontozygomatic suture. The table lists modified and not modified length implants. The analysis of complications (see below) showed no difference in risk of complications after adaptations.

**Table 3:** Osteosynthesis implant usage (Medartis Modus Midface®).

Plate	Holes	Full length	Shortended	Total	% of total implants (%)
2.0 mm straight, without bar	4	500	0	500	37.6
1.5 mm without bar	4	130	1	131	9.9
1.5 mm without bar	8	84	36	120	9.0
1.5 mm without bar	6	99	15	114	8.6
L/Y/T-shaped 1.5 mm	Any	102	10	112	8.4
2.0 mm with bar	4	72	0	72	5.4
2.0 mm without bar	4	52	0	52	3.9
Orbital mesh, 0.1 mm	–	0	46	46	3.5
Other	–	–	–	182	13.7
Total	–	–	–	1329	100

The complications are rare: we observed a total of 58 patients with severe complications. There were no lethal or life-threatening ones observed. The list of the complications e.g. their combinations are listed in Table 4. The majority of the complications are related to the procedure and a few of them are related to the osteosynthesis implants. In case of midface fractures, the complication rate (Clavien-Dindo Class II–IV) was 2.02%.

**Table 4:** List of the complications in different regions and their proportion to all midface complications.

Complication	Complication site	Total	% of total (%)
Excessive scarring	Zygomatic bone	12	20.7
	Naso-orbito-ethmoid		
Wound healing problem	Zygomatic bone	8	13.8
	Le fort I		
Insufficient reposition	Zygomatic bone	8	13.8
	Le fort I		
Infection	Le fort I	5	8.6
	Zygomatic bone		
Screw loosening	Zygomatic bone	4	6.9
	Le fort II		
Osteointegration	Zygomatic bone	3	5.2
Hypesthesia	Zygomatic bone	3	5.2
Postoperative retrobulbar hematoma	Orbital floor	1	1.7
Pansinusitis	Zygomatic bone	1	1.7

## Discussion

The patient treatment protocol follows the AO guidelines^24,25^. This is the first study—as per authors’ information—that reports clinical results with the Medartis Modus® system. As new device designs and materials are marketed since osteosynthesis became a proven method^34^, re-evaluation of the novel developments is to be examined. This study is a retrospective assessment of 11 years of the treatment period in 2868 patients.

Most fractures are nasal bone, zygomatic bone and orbital floor fractures. Generally, the etiology and fracture distribution are similar to those in the international literature^3,7,35–37^. Some authors report more mandibular fractures than midfacial ones, in our registry midface fractures dominate (2868 vs. 1096 patients)^38^. The complication rate was 2.2%, which is below the international average^22,39,40^. The average time to the first surgery and the total inpatient stay is also shorter than it was reported by other authors^19,20,41,42^. This also helps to reduce the complication rate and is only possible with an experienced and well-managed team. In our opinion, immediate interdisciplinary diagnosis and therapy planning, early surgery and adequate postoperative care, including sufficient intensive care units and short hospital stay help reducing the overall complication rates. The midface is more stable and biomechanically less stressed than for example the mandibular angle or paramedian area. The aforementioned improvement in three-dimensional diagnostics and implant quality allows clearly easier surgery for orbital injuries, too. All these explain the better results in midface area than the overall complication rates, too. The osteosynthesis implants showed excellent stability, failures were in just a few cases reported. Most of these cases have not lead to a non-union, pseudoarthrosis, or osteomyelitis. Thus, the safety and efficacy of the methods and materials that are used in our surgery are proven.

The high incidence rate in young male and old female patients is alarming. At young age interpersonal violence and road traffic accidents are important etiologies. In older females, most cases are related to falls, less to road traffic accidents. Interpersonal violence is much rarely reported. Alcoholic influence is in both cases an important factor but in people older than 75 y.o. most falls are related to poor medical conditions. It is important, to apply more prevention in both patient groups to decrease the occurrence of facial fractures. As the number of admissions varies around yearly 260 ± 40 cases during the whole study period with a slightly worsening trend. In the whole registry, the yearly number of underage patients is decreasing, while in the elderly, the situation is getting worse. In younger men and older females more specific preventive work should be performed in order to reduce injury rates and consequences.

The limitation of the study is its retrospective nature. Some data, including radiographs, was missing. This was < 1% and missing data was mostly reproducible from the existing parts of the patient documentation. This retrospective nature of the study also carries advantages. The concept is the same during the study, the operations are carried out or supervised by the same, experienced surgeons. The treatment follows the above concept, but is highly individualized, thus a bias by using non-adaptable study protocols is reduced.

In conclusion, we can state, that the diagnostic and treatment protocols are appropriate, both these and sufficient experience supports good results and low complication rates. The novel osteosynthesis implants are safe and the surgeries can be performed on time.

The study covers a long time frame that is among the longest periods that have been reviewed. Another limitation of this study is that it is a monocentric one. A multicentric, prospective extension of this study (for example in Germany or in Europe) would bring still more scientific value. This requires, however, continuous efforts at all research centers that can increase long-term bias due to non-reporting cases. Due to the high work load with analyzing all radiographs and thorough monitoring, the study duration was long, the results, however, more standardized.

In conclusion, this study has shown that the three most common fractures of the midface area, nasal bone fractures, zygomatic bone fractures and orbital floor fractures (40.1%, 23.9% and 20.1% respectively) make 84.1% of all midface fractures. Due to improvement of diagnostics, therapy and materials used for surgery, the complication rate can be decreased to 2.2%, which is well below the internationally reported complication rates. This proves our hypothesis that the current guidelines and workflows contribute to improvement to patient safety and better treatment outcome.

## Data Availability

Data availability is ruled by the Data Protection laws of the European Union, the Federal Republic of Germany, the State North-Rhine-Westphalia and the regulations of Dortmund General Hospital. Data is therefore on purpose on-site available.
